# 4-Bromo-8-methoxy­quinoline

**DOI:** 10.1107/S1600536808014591

**Published:** 2008-05-21

**Authors:** Neil Vasdev, Padmakar V. Kulkarni, Alan A. Wilson, Sylvain Houle, Alan J. Lough

**Affiliations:** a PET Centre, Centre for Addiction and Mental Health and Department of Psychiatry, University of Toronto, 250 College Street, Toronto, Ontario, Canada M5T 1R8; b Department of Radiology, The University of Texas, Southwestern Medical Center at Dallas, 5323 Harry Hines Blvd, Dallas, Texas 75390, USA; c Department of Chemistry, University of Toronto, 80 St. George Street, Toronto, Ontario, Canada M5S 3H6

## Abstract

The non-H atoms of the title mol­ecule, C_10_H_8_BrNO, are essentially coplanar. In the crystal structure, mol­ecules are linked by weak inter­molecular C—H⋯π(arene) inter­actions, forming one-dimensional chains along the *a* axis.

## Related literature

For related literature, see: Michael (2008 ▶); Kulkarni *et al.* (2006 ▶); Irving & Pinnington (1957 ▶).
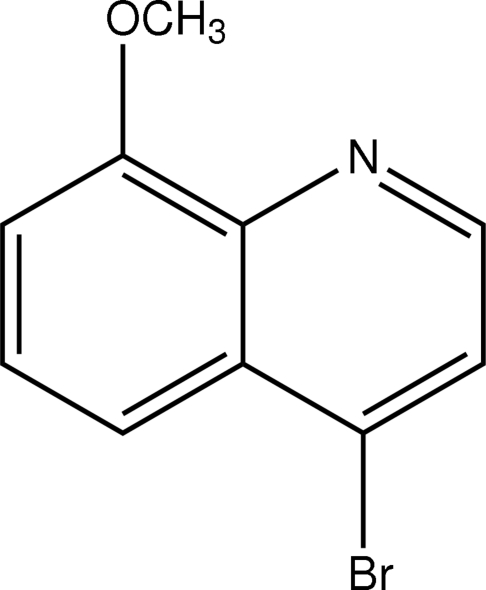


## Experimental

### 

#### Crystal data

C_10_H_8_BrNO
*M* *_r_* = 238.08
Orthorhombic, 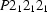

*a* = 5.1615 (1) Å
*b* = 12.1337 (6) Å
*c* = 14.2436 (7) Å
*V* = 892.05 (6) Å^3^
*Z* = 4
Mo *K*α radiation
μ = 4.56 mm^−1^
*T* = 150 (1) K
0.30 × 0.12 × 0.11 mm

#### Data collection

Nonius KappaCCD diffractometer
Absorption correction: multi-scan (*SORTAV*; Blessing 1995 ▶) *T*_min_ = 0.545, *T*_max_ = 0.607
6134 measured reflections
2026 independent reflections
1872 reflections with *I* > 2σ(*I*)
*R*_int_ = 0.035

#### Refinement

*R*[*F* ^2^ > 2σ(*F* ^2^)] = 0.028
*wR*(*F* ^2^) = 0.059
*S* = 1.01
2026 reflections
120 parameters
H-atom parameters constrained
Δρ_max_ = 0.38 e Å^−3^
Δρ_min_ = −0.40 e Å^−3^
Absolute structure: Flack (1983 ▶), 815 Friedel pairs
Flack parameter: −0.017 (11)

### 

Data collection: *COLLECT* (Nonius, 2002 ▶); cell refinement: *DENZO-SMN* (Otwinowski & Minor, 1997 ▶); data reduction: *DENZO-SMN*; program(s) used to solve structure: *SIR92* (Altomare *et al.*, 1994 ▶); program(s) used to refine structure: *SHELXTL* (Sheldrick, 2008 ▶); molecular graphics: *PLATON* (Spek, 2003 ▶) and *SHELXTL*; software used to prepare material for publication: *SHELXTL*.

## Supplementary Material

Crystal structure: contains datablocks global, I. DOI: 10.1107/S1600536808014591/pv2082sup1.cif

Structure factors: contains datablocks I. DOI: 10.1107/S1600536808014591/pv2082Isup2.hkl

Additional supplementary materials:  crystallographic information; 3D view; checkCIF report

## Figures and Tables

**Table 1:** Hydrogen-bond geometry (Å, °)

*D*—H⋯*A*	*D*—H	H⋯*A*	*D*⋯*A*	*D*—H⋯*A*
C10—H10*A*⋯*Cg*^i^	0.98	2.66	3.531 (3)	148

Symmetry code: (i) 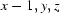. *Cg* is the centroid of the C4–C9 ring.

**Fig. 1.**
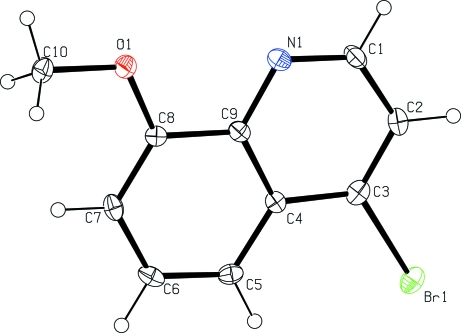
Molecular structure showing 30% probability displacement ellipsoids (arbitrary spheres for H atoms).

**Fig. 2.**
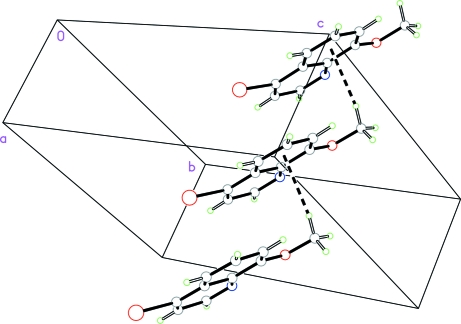
Part of the crystal structure showing weak C—H···π(arene) interactions as dashed lines.

## supplementary crystallographic information

### Comment

Quinoline derivatatives are established chelating agents and also have applications as precursors for pesticides and pharmaceuticals (Michael, 2008). Our laboratories are pursuing the development of radiohalogenated 8-hydroxyquinoline derivatives for positron emission tomography (PET) and single photon emission computed tomography (SPECT), specifically to image extracellular glial deposition of amyloid plaque protein in Alzheimer's disease and matrix metalloproteinases in tumours (Kulkarni *et al.*, 2006). 4-Bromo-8-methoxyquinoline, first reported by Irving & Pinnington (1957) may be used as a precursor for radiohalogenation reactions to prepare labelled 8-hydroxyquinoline-based PET or SPECT radiopharmaceuticals. To our surprise, neutral compounds bearing a 4-halogen substituted, 8-phenoxyquinoline core have not yet been studied by single-crystal X-ray crystallography. In the present study we report the crystal structure of the title compound at 150 K.

The non-hydrogen atoms of title molecule (Fig. 1), C_10_H_8_BrNO, are essentially co-planar (r.m.s. deviation of all non-H atoms = 0.0242 Å). In the crystal structure, molecules are linked by weak intermolecuar C—H···π(arene) interactions to form one-dimensional chains along the *a* axis (Fig. 2). There are no other hydrogen bonds or π···π stacking interactions.

### Experimental

X-ray quality crystals were obtained by evaporation of a solution of the title compound (ECA International Corporation, Palatine, Illinois, USA) in chloroform.

### Refinement

H atoms were placed in calculated positions with C—H = 0.95Å (aryl) and 0.98Å (methyl) and were included in the refinement in the riding-model approximation with *U*_iso_(H) = 1.2*U*_eq_(C) or *U*_iso_(H) = 1.5*U*_eq_(C) for methyl H atoms.

### Crystal data

**Table:**

C_10_H_8_BrNO	*F*_000_ = 472
*M**_r_* = 238.08	*D*_x_ = 1.773 Mg m^−^^3^
Orthorhombic, *P*2_1_2_1_2_1_	Mo *K*α radiation λ = 0.71073 Å
Hall symbol: P 2ac 2ab	Cell parameters from 6134 reflections
*a* = 5.1615 (1) Å	θ = 2.9–27.5º
*b* = 12.1337 (6) Å	µ = 4.56 mm^−^^1^
*c* = 14.2436 (7) Å	*T* = 150 (1) K
*V* = 892.05 (6) Å^3^	Needle, colourless
*Z* = 4	0.30 × 0.12 × 0.11 mm

### Data collection

**Table:**

Nonius KappaCCD diffractometer	2026 independent reflections
Radiation source: fine-focus sealed tube	1872 reflections with *I* > 2σ(*I*)
Monochromator: graphite	*R*_int_ = 0.036
Detector resolution: 9 pixels mm^-1^	θ_max_ = 27.5º
*T* = 150(2) K	θ_min_ = 2.9º
φ scans and ω scans with κ offsets	*h* = −5→6
Absorption correction: multi-scan(SORTAV; Blessing 1995)	*k* = −14→15
*T*_min_ = 0.545, *T*_max_ = 0.607	*l* = −18→18
6134 measured reflections	

### Refinement

**Table:**

Refinement on *F*^2^	H-atom parameters constrained
Least-squares matrix: full	*w* = 1/[σ^2^(*F*_o_^2^) + (0.0306*P*)^2^ + 0.0333*P*] where *P* = (*F*_o_^2^ + 2*F*_c_^2^)/3
*R*[*F*^2^ > 2σ(*F*^2^)] = 0.028	(Δ/σ)_max_ = 0.001
*wR*(*F*^2^) = 0.059	Δρ_max_ = 0.38 e Å^−^^3^
*S* = 1.01	Δρ_min_ = −0.40 e Å^−^^3^
2026 reflections	Extinction correction: SHELXTL (Sheldrick, 2008), Fc^*^=kFc[1+0.001xFc^2^λ^3^/sin(2θ)]^-1/4^
120 parameters	Extinction coefficient: 0.0062 (8)
Primary atom site location: structure-invariant direct methods	Absolute structure: Flack (1983), 815 Friedel pairs
Secondary atom site location: difference Fourier map	Flack parameter: −0.017 (11)
Hydrogen site location: inferred from neighbouring sites	

### Special details

**Table:**

Geometry. All e.s.d.'s (except the e.s.d. in the dihedral angle between two l.s. planes) are estimated using the full covariance matrix. The cell e.s.d.'s are taken into account individually in the estimation of e.s.d.'s in distances, angles and torsion angles; correlations between e.s.d.'s in cell parameters are only used when they are defined by crystal symmetry. An approximate (isotropic) treatment of cell e.s.d.'s is used for estimating e.s.d.'s involving l.s. planes.
Refinement. Refinement of *F*^2^ against ALL reflections. The weighted *R*-factor *wR* and goodness of fit *S* are based on *F*^2^, conventional *R*-factors *R* are based on *F*, with *F* set to zero for negative *F*^2^. The threshold expression of *F*^2^ > σ(*F*^2^) is used only for calculating *R*-factors(gt) *etc*. and is not relevant to the choice of reflections for refinement. *R*-factors based on *F*^2^ are statistically about twice as large as those based on *F*, and *R*- factors based on ALL data will be even larger.

### Fractional atomic coordinates and isotropic or equivalent isotropic displacement parameters (Å^2^)

**Table:**

	*x*	*y*	*z*	*U*_iso_*/*U*_eq_	
Br1	1.02034 (5)	0.36147 (2)	0.509948 (18)	0.02940 (11)	
O1	0.1007 (3)	0.62674 (16)	0.72708 (14)	0.0259 (4)	
N1	0.4260 (4)	0.64140 (17)	0.58326 (14)	0.0233 (4)	
C1	0.5884 (5)	0.6481 (2)	0.51287 (18)	0.0278 (6)	
H1A	0.5819	0.7126	0.4752	0.033*	
C2	0.7717 (5)	0.5671 (2)	0.48878 (19)	0.0268 (6)	
H2A	0.8842	0.5768	0.4366	0.032*	
C3	0.7833 (5)	0.4746 (2)	0.54217 (19)	0.0239 (6)	
C4	0.6190 (5)	0.4611 (2)	0.62176 (17)	0.0188 (5)	
C5	0.6239 (5)	0.3694 (2)	0.68244 (18)	0.0232 (6)	
H5A	0.7429	0.3109	0.6718	0.028*	
C6	0.4556 (5)	0.3650 (2)	0.75705 (17)	0.0243 (6)	
H6A	0.4624	0.3038	0.7986	0.029*	
C7	0.2739 (5)	0.4487 (2)	0.77325 (18)	0.0235 (6)	
H7A	0.1558	0.4424	0.8241	0.028*	
C8	0.2656 (5)	0.5400 (2)	0.71586 (17)	0.0195 (5)	
C9	0.4408 (5)	0.54916 (19)	0.63810 (16)	0.0199 (5)	
C10	−0.0731 (5)	0.6201 (2)	0.80535 (18)	0.0271 (7)	
H10C	−0.1784	0.6872	0.8083	0.041*	
H10D	0.0269	0.6126	0.8635	0.041*	
H10A	−0.1865	0.5559	0.7978	0.041*	

### Atomic displacement parameters (Å^2^)

**Table:**

	*U*^11^	*U*^22^	*U*^33^	*U*^12^	*U*^13^	*U*^23^
Br1	0.02472 (15)	0.03108 (16)	0.03239 (16)	0.00303 (10)	0.00306 (11)	−0.00756 (11)
O1	0.0264 (10)	0.0255 (11)	0.0259 (10)	0.0030 (8)	0.0047 (7)	0.0004 (8)
N1	0.0281 (10)	0.0212 (11)	0.0206 (11)	−0.0009 (9)	−0.0021 (8)	0.0001 (10)
C1	0.0339 (13)	0.0259 (14)	0.0237 (14)	−0.0019 (10)	0.0030 (10)	0.0087 (14)
C2	0.0265 (12)	0.0299 (14)	0.0240 (14)	−0.0065 (10)	0.0049 (12)	0.0001 (13)
C3	0.0215 (13)	0.0257 (14)	0.0246 (15)	−0.0015 (10)	−0.0025 (10)	−0.0078 (12)
C4	0.0210 (13)	0.0184 (13)	0.0169 (13)	−0.0037 (9)	−0.0018 (9)	−0.0016 (11)
C5	0.0224 (12)	0.0215 (14)	0.0257 (14)	0.0016 (11)	−0.0064 (10)	−0.0019 (12)
C6	0.0328 (15)	0.0176 (13)	0.0225 (13)	−0.0037 (13)	−0.0074 (11)	0.0035 (11)
C7	0.0257 (14)	0.0258 (15)	0.0191 (14)	−0.0088 (11)	0.0018 (11)	−0.0010 (11)
C8	0.0208 (13)	0.0185 (13)	0.0193 (13)	−0.0003 (10)	−0.0021 (10)	−0.0014 (11)
C9	0.0207 (12)	0.0204 (13)	0.0186 (12)	−0.0042 (10)	−0.0046 (10)	0.0010 (10)
C10	0.0240 (14)	0.0314 (17)	0.0258 (15)	0.0015 (12)	0.0047 (11)	−0.0028 (12)

### Geometric parameters (Å, °)

**Table:**

Br1—C3	1.895 (2)	C4—C9	1.429 (3)
O1—C8	1.362 (3)	C5—C6	1.374 (4)
O1—C10	1.433 (3)	C5—H5A	0.9500
N1—C1	1.309 (3)	C6—C7	1.402 (4)
N1—C9	1.367 (3)	C6—H6A	0.9500
C1—C2	1.407 (4)	C7—C8	1.378 (4)
C1—H1A	0.9500	C7—H7A	0.9500
C2—C3	1.357 (3)	C8—C9	1.434 (3)
C2—H2A	0.9500	C10—H10C	0.9800
C3—C4	1.425 (3)	C10—H10D	0.9800
C4—C5	1.409 (4)	C10—H10A	0.9800
			
C8—O1—C10	116.0 (2)	C5—C6—H6A	119.3
C1—N1—C9	116.9 (2)	C7—C6—H6A	119.3
N1—C1—C2	125.0 (2)	C8—C7—C6	120.4 (2)
N1—C1—H1A	117.5	C8—C7—H7A	119.8
C2—C1—H1A	117.5	C6—C7—H7A	119.8
C3—C2—C1	118.1 (2)	O1—C8—C7	124.8 (2)
C3—C2—H2A	121.0	O1—C8—C9	115.1 (2)
C1—C2—H2A	121.0	C7—C8—C9	120.0 (2)
C2—C3—C4	121.0 (2)	N1—C9—C4	123.7 (2)
C2—C3—Br1	119.4 (2)	N1—C9—C8	118.0 (2)
C4—C3—Br1	119.58 (19)	C4—C9—C8	118.3 (2)
C5—C4—C3	124.6 (2)	O1—C10—H10C	109.5
C5—C4—C9	120.1 (2)	O1—C10—H10D	109.5
C3—C4—C9	115.3 (2)	H10C—C10—H10D	109.5
C6—C5—C4	119.6 (3)	O1—C10—H10A	109.5
C6—C5—H5A	120.2	H10C—C10—H10A	109.5
C4—C5—H5A	120.2	H10D—C10—H10A	109.5
C5—C6—C7	121.5 (2)		

### Hydrogen-bond geometry (Å, °)

**Table:**

*D*—H···*A*	*D*—H	H···*A*	*D*···*A*	*D*—H···*A*
C10—H10A···Cg^i^	0.98	2.66	3.531 (3)	148

Symmetry codes: (i) *x*−1, *y*, *z*.

## References

[bb1] Altomare, A., Cascarano, G., Giacovazzo, C., Guagliardi, A., Burla, M. C., Polidori, G. & Camalli, M. (1994). *J. Appl. Cryst.* **27**, 435.

[bb2] Blessing, R. H. (1995). *Acta Cryst.* A**51**, 33–38. 10.1107/s0108767394005726 7702794

[bb3] Flack, H. D. (1983). *Acta Cryst.* A**39**, 876–881.

[bb4] Irving, H. & Pinnington, A. R. (1957). *J. Chem. Soc.* pp. 285–290.

[bb5] Kulkarni, P., Arora, V., Bennett, M., Roney, C., Partridge, K., Lewis, M., Antich, P. & Bonte, F. (2006). *J. Nucl. Med.* **47**, 509P–510P.

[bb6] Michael, J. P. (2008). *Nat. Prod. Rep.* **25**, 166–187. 10.1039/b612168n 18250901

[bb7] Nonius (2002). *COLLECT* Nonius BV, Delft, The Netherlands.

[bb8] Otwinowski, Z. & Minor, W. (1997). *Methods in Enzymology*, Vol. 276, *Macromolecular Crystallography*, Part A edited by C. W. Carter Jr & R. M. Sweet pp. 307–326. London: Academic press.

[bb9] Sheldrick, G. M. (2008). *Acta Cryst.* A**64**, 112–122. 10.1107/S0108767307043930 18156677

[bb10] Spek, A. L. (2003). *J. Appl. Cryst.* **36**, 7–13.

